# Does Anxiety Affect the Clock-Drawing Task in Patients in a Memory Clinic? A Clinical Correlation Study

**DOI:** 10.1093/geroni/igaa057.910

**Published:** 2020-12-16

**Authors:** Hamed Khachan, Mahak Kanjolia, Anil Nair

**Affiliations:** Alzheimer’s Disease Center, Quincy, Massachusetts, United States

## Abstract

Background: It is unknown if anxiety levels affect performance on clock drawing test (CDT) in memory clinic patients. Method: We performed a retrospective analysis of memory clinic patients in the south shore of Boston from 2010 to 2019. We correlated anxiety screen data (GAD7) to CDT scores, based on contour, numbers, and hands placement. Univariate analyses used Spearman correlation. A multivariate regression model analzed GAD7 to covariates of CDT, age, sex, and race. Hypothesis : We hypothesized a positive correlation between anxiety levels scored by the GAD7 and CDT. Results: 994 patients in the memory clinic between 2010-2020 had analyzable data. Patients were 58.6% female, 84.6 % White. Mean age was 70.1±14.4, CDT 1.84±1.04. CDT score correlated significantly to race (⍴=-0.16, p< 0.001), age (⍴=-0.28, p<0.001), gender (⍴=0.05, p=0.16), but not GAD7 (⍴=0.05, p=0.27). Multivariate model confirmed the lack of association of anxiety scores to CDT (□= 0.08, p=0.78). GAD7 scores correlated to female gender (□= -1.16, p=0.04). Conclusions: CDT scores were not affected by anxiety as measured on GAD7 scores. However, a positive correlation was shown on anxiety scores in females to CDT completion.

